# Impact of COVID-19 pandemic on insomnia and sleep efficiency in parents and caregivers of young children

**DOI:** 10.3389/frsle.2023.1212784

**Published:** 2023-07-04

**Authors:** Nana Jiao, Keenan A. Pituch, Megan E. Petrov

**Affiliations:** Edson College of Nursing and Health Innovation, Arizona State University, Phoenix, AZ, United States

**Keywords:** parental sleep, children <6 years, sleep quality, sleep patterns, COVID-19, crisis

## Abstract

**Introduction:**

During the COVID-19 pandemic, sleep problems were highly prevalent. However, few studies assess sleep quality among parents and caregivers with young children. This study aimed to describe the impacts of the COVID-19 pandemic on sleep patterns among parents and caregivers with young children (<6 years) and identify the factors associated with insomnia and sleep efficiency.

**Methods:**

An internet sample of 136 caregivers (age: 35 ± 9.7 y, 70.6% female) were recruited internationally from May 21, 2020 to July 1, 2020. Participants completed the Center for Epidemiological Studies–Depression Scale-10 (CES-D-10), Sleep Hygiene and Practices Scale (SHPS), Coronavirus Impact Scale (CIS), Insomnia Severity Index (ISI), and sleep patterns prior to and during the pandemic. Hierarchical regressions were conducted to examine the factors associated with insomnia and sleep efficiency.

**Results:**

Nearly 40% of the caregivers reported household incomes <$10,000. More than half reported clinical levels of depressive symptoms (59.2%) and low sleep efficiency (65.8%). Approximately 90% reported that their sleep-wake routine was altered with delayed bedtime and midpoint, and more naps and nightmares. Almost half (51.5%) were experiencing clinically meaningful insomnia symptoms. Greater insomnia symptom severity was independently associated with lower income, greater depressive symptoms, poor sleep hygiene behaviors, altered sleep-wake routine, and greater COVID-related disruptions in daily life. The predictors associated with poor sleep efficiency during the pandemic were lower income and poor sleep efficiency before the pandemic.

**Discussion:**

The study highlighted the factors associated with insomnia and poor sleep efficiency during the COVID-19 pandemic. Interventions are needed to support caregivers' sleep during global crises.

## Introduction

The acute stages of the novel coronavirus (COVID-19) pandemic led to drastic and stressful changes in daily life, such as quarantine, social isolation, unemployment, and disrupted work-life routines. All these factors, along with distress and fear of getting infected, were associated with sleep problems with a high prevalence ranging from 17.4 to 57.2% in the general population (Cellini et al., 2020; Huang and Zhao, 2020). Moreover, COVID-19 pandemic-related sleep problems appeared to be persisting over time despite greater disease control and an increased intake rate of vaccinations (Li et al., 2021). Chronic poor sleep was associated with adverse long-term health outcomes (Khan and Aouad, 2017; Pigeon et al., 2017), especially among COVID-19 patients (Papagiouvanni et al., 2022). A recent systematic review on sleep and vaccination of influenza and hepatitis indicated a positive correlation between sleep and immune response (Rayatdoost et al., 2022). Sufficient and quality sleep was reported to double the antigen-specific immune response (Garbarino and Scoditti, 2021; Kow and Hasan, 2021).

During the COVID-19 pandemic, the sleep quality of caregivers with older children and adolescents was compromised (Peltz et al., 2020; Cellini et al., 2021; El-Osta et al., 2021; Ruppanner et al., 2021; Wearick-Silva et al., 2022), including later bedtime, poor sleep latency, and poor sleep efficiency (SE). However, reports among caregivers of young children (< 6 y) were mixed and few in number. Cumulative sleep loss among parents with young children is common (Falkingham et al., 2022) such that prior to the pandemic they experienced reduced nightly sleep duration in comparison to parents with older children (Hagen et al., 2013). Children's sleep may partly explain the deterioration of parental sleep (Sinai and Tikotzky, 2012; Rönnlund et al., 2016; Staples et al., 2019). Until the age of three, parents frequently report their child waking up at least once a week, which may disturb their sleep patterns (Scher et al., 2004). Poorer parental sleep is also linked to parenting stress during the first year postpartum (Sinai and Tikotzky, 2012). However, whether the pandemic further exacerbated these pre-pandemic associations is unclear. One cross-sectional study found 60% of caregivers of young children (< 6 y) reported worse sleep quality during the pandemic, and a higher clinical prevalence of insomnia symptoms compared to the pre-pandemic period (23 vs. 11%) (Zreik et al., 2020). These greater maternal insomnia symptoms were positively associated with their child's sleep latency, number of night awakenings, and wake time after sleep onset (Zreik et al., 2020). Further, another longitudinal study reported longer sleep latency and similar nocturnal sleep time among parents of young children as compared with the pre-pandemic period (Shinomiya et al., 2021). Yet, another longitudinal study indicated that there was no apparent change in parents of young children's sleep quality when compared with the pre-pandemic period (Kahn et al., 2021).

Multiple factors may have contributed to these mixed findings on caregiver sleep health, such as differences in pandemic-related impacts on daily life, mental wellbeing, socioeconomic status, and sleep-related behaviors (Bartoszek et al., 2020; Cellini et al., 2020; Zreik et al., 2020; Sosso et al., 2021; Peltz et al., 2023). Around 70% of caregivers reported that the COVID-19 pandemic consequently disrupted daily life, increased stress levels (American Psychological Association, 2020), and affected health behaviors (Janssen et al., 2020). Caregivers of young children faced the additional responsibilities of dealing with fluctuating stay-at-home orders and childcare, balancing work and family at home, and making decisions regarding the health and vaccination of their children (Adams, 2020; Calvano et al., 2021; Prime et al., 2022). Further, the mental wellbeing of parents and caregivers deteriorated during the pandemic (Goldberg et al., 2021) including heightened depressive symptoms (Lyttelton et al., 2020; Collins et al., 2021; Johnson et al., 2021). They worried about family health and broader global issues such as the national economy and impacts on their family's financial stability. Additionally, around 40–54.2% of parents reported reduced family income due to the COVID-19 crisis (Karpman et al., 2020; Zreik et al., 2020). However, few studies examined the predictors of poor sleep among parents and caregivers with young children during the COVID-19 pandemic.

This study aims to describe changes in sleep patterns among parents and caregivers with young children prior to and during the early stages of the COVID-19 pandemic and identify factors associated with insomnia symptoms and sleep efficiency. Based on the social-ecological model of sleep health (Grandner, 2019), we hypothesized that poorer mental wellbeing (individual level), poorer sleep-related behaviors (individual level), lower socioeconomic status (social level), and greater pandemic-related impacts on daily life (societal level), would independently contribute to poorer sleep quality.

## Materials and methods

### Study design and participants

In this international cross-sectional study, participants completed a 25-min online survey distributed through the Qualtrics platform from May 21, 2020 to July 1, 2020. During this period, many countries were either imposing stay-at-home orders or gradual economic reopening, including the Americas, Middle East and South Asia, and parts of Africa. The Institutional Review Board of Arizona State University approved the study, and electronic consent was provided.

The only eligibility criterion for the parent study was that the participant could read and understand English. Recruitment advertisements included: (1) mainly, paid Facebook/Instagram distributed globally; (2) local institutional email listservs and banner advertisements; and (3) word-of-mouth. More detailed study information has been published (Petrov et al., 2021). In total, 991 global participants completed the survey. Of those, a subsample of 136 participants reported that they were parents and caregivers of young children. All analyses were performed on this subsample.

### Measures

#### Sociodemographic and socioeconomic information

Sample characteristics included: age; sex (female/male); race (White, Asian, Black, American Indian/Native American, Pacific Islander/Native Hawaiian, or others/mixed race); education (< bachelor degree, bachelor degree or above); household income in U.S. dollars (< $10,000, $10,000–49,999, or ≥$50,000); number of children (< 6 y) in the household; country (US, or others). All participants responded whether their region was “currently imposing stay-at-home/quarantine measures” (“yes,” “no,” or “the authorities stopped and/or relaxed stay-at-home quarantine measures in my region recently”).

#### Depressive symptoms

The Center for Epidemiological Studies-Depression Scale-10 (CES-D-10, 10-item, range: 0-30, cutoff: 10) assessed how often participants experience each depressive symptom in the past week (i.e., < 1, 1–2, 3–4, or 5–7 days) (Andresen et al., 1994). The CES-D-10 is validated among parents, with good internal consistency (Ferro and Speechley, 2013). Greater scores indicate more severe depressive symptoms.

#### Sleep-wake routine and sleep-related health behaviors

Participants were queried regarding whether their sleep-wake routine during quarantine/stay-at-home orders was changed and consistent with their personal preference or “body clock” (yes, and still not consistent; yes, and somewhat more consistent; yes, and much more consistent; N/A, my sleep/wake routine has not changed and is consistent with my personal preference). Further, participants completed the Sleep Hygiene and Practices Scale (SHPS, 30-item), a 6-point Likert scale with greater values indicating a greater frequency of engaging in behaviors that may impinge on sleep (Yang et al., 2010). The SHPS is comprised of four subscales, including behaviors related to homeostatic sleep drive and/or circadian sleep-wake rhythms (sleep scheduling/timing), arousal-associated activities (enhancing arousal through the promotion of anxiety and/or conditioned arousal with sleep-related cues), eating/drinking behaviors prior to sleep, and the sleep environment.

#### Overall impact of COVID-19

The Coronavirus Impact Scale (CIS), a validated 12-item scale with good internal consistency among families with children (Stoddard et al., 2023), examined multiple daily life aspects affected by the COVID-19 pandemic. Responses range from “no change” to “severe” on a 4-point Likert scale. The first eight items included daily routines, employment status, food access, access to medical healthcare, access to mental healthcare, access to social support from extended family and non-family, experiences of pandemic-related stress, and family stress and discord (Stoddard et al., 2023). Responses to the eight items are summed, yielding a total impact score ranging from 0 to 24, with greater scores indicating a greater impact on daily life.

#### Sleep outcomes

Participants responded to questions regarding their typical bedtime, wake time, sleep-onset latency (SOL, min), wake time after sleep onset (WASO, min), and nightmares and naps/week both prior (retrospectively) and during the pandemic. The following variables were derived: time-in-bed (TIB = wake time-bedtime), nocturnal total sleep time (nTST = TIB-SOL-WASO), sleep efficiency (SE% = [nTST/TIB] ^*^ 100%, low sleep efficiency: < 85 %), and sleep midpoint in clock time ([TIB/2] + bedtime). SE% was obtained from the assessment of sleep during the pandemic.

Insomnia Severity Index (ISI), a validated 7-item index with adequate internal consistency among parents (Urfer-Maurer et al., 2017), evaluated self-perceived insomnia severity over the past 2 weeks. Responses range from “no problem” to “very severe” on a 5-point Likert scale, yielding a total score ranging from 0 to 28. A cutoff score of 10 is meaningful in the general community (Morin et al., 2011).

### Data analyses

Paired samples *t*-tests were used to compare sleep patterns before and during the COVID-19 pandemic. Bivariate associations of a priori hypothesized factors to predict ISI scores and sleep efficiency were conducted with one-way ANOVAs (Supplementary Table 1) or Pearson correlation (Supplementary Table 2). To determine which predictors were independently associated with ISI and sleep efficiency, a hierarchical regression procedure was used. In considering 1 *df* per 10 participants, no more than 12 and 10 independent variables were included in the regression analysis for ISI (*n* = 121) and sleep efficiency (*n* = 108), respectively, with these sample sizes produced by the use of pairwise deletion for the regression analysis. As such, the predictors we included in the regression models for ISI and SE were those that were (a) significantly associated with the respective outcome (*p* < 0.05 and η^2^ ≥ 0.06 in one-way ANOVAs) or (b) whose correlation coefficient exceeded a magnitude of 0.3 (i.e., *r* ≥ |0.3| in Pearson correlation). A priori hierarchical blocks of variables were entered into the model. The 1st block was demographic information. Mental health variables were entered into the 2nd block. The 3rd block covered all the sleep-related variables, such as SHPS subscales, the change in the sleep-wake routine, and sleep efficiency before the pandemic (for sleep efficiency during the pandemic only). Lastly, CIS total score was added into the 4th block. Categorical variables were dummy coded, including sex, household income, education, and change in sleep-wake routine.

## Results

### Descriptive results

Table 1 displays the sample's characteristics. Participants' average age was 35 years old and were mostly female (70.6%). A considerable portion reported low household incomes < $10,000 (35.1%). More than one-third (37.5%) had more than two young children in the household. Most of the sample reported clinical levels of depressive symptoms (59.2%, cutoff: 10) and low sleep efficiency (65.8%). Nearly half experienced insomnia symptoms (51.5%, cutoff: 10).

**Table 1:** Descriptive characteristics of participants (*n* = 136).

**Socio-demographic characteristics**	***n* (%)**	**Mean (SD)**
Age (mean, SD)	135	35 (9.7)
Education	136	
Below bachelor	37 (27.2)	
Bachelor or above	99 (72.8)	
Biological sex	136	
Male	40 (29.4)	
Female	96 (70.6)	
Have children < 6 y in the household	136	
1	85 (62.5)	
≥2	51 (37.5)	
Household income (USD)	134	
< $10,000	47 (35.1)	
$10,000–$49,999	38 (28.4)	
≥$50,000	49 (36.6)	
Race	134	
Asian	58 (43.3)	
White or others	76 (56.7)	
Stay-at-home/quarantine	136	
Yes	57 (41.9)	
No or relaxed quarantine measures recently	79 (58.1)	
Country	136	
US	53 (39)	
Other countries	83 (61)	
**Mental health/sleep outcomes and COVID-19-related impacts**	***n*** **(%)**	**Mean (SD)**
CES-D-10 (range: 0–30)	130	12.3 (6.5)
SHPS total score (range: 30–180)	128	79.8 (19.6)
Sleep scheduling behaviors (range: 7–42)	132	23.2 (6.8)
Arousal-associated behaviors (range: 9–54)	132	25.4 (7.3)
Eating/drinking behaviors (range: 6–36)	131	11.9 (4.1)
Sleep environment (range: 8–48)	130	19 (7.4)
Whether quarantine sleep-wake routine was changed and consistent with their personal preference	128	
Yes, not consistent with their personal preference	62 (48.4)	
Yes, somewhat more consistent with their personal preference	29 (22.7)	
Yes, much more consistent with their personal preference	22 (17.2)	
No change and consistent with their personal preference	15 (11.7)	
CIS total score	134	11.2 (4.6)
ISI (range: 0–28)	136	10.4 (6.6)
SE% during the pandemic	117	74.8 (18.7)

Table 2 displays the sleep patterns before and during the COVID-19 pandemic, and paired *t*-tests for each sleep variable. There were significant changes in all sleep variables except TIB and nTST. Overall, the sample delayed their bedtime by nearly 1.5 h later, delayed midpoint of sleep by about over 1 h, experienced a 9.48% reduction in sleep efficiency, and experienced increases in weekly nightmares (+1.5) and naps (+1.1).

**Table 2:** Sleep change characteristics with paired samples *t*-tests comparing sleep patterns before and during the COVID-19 pandemic.

	**Variables**	**M(SD)**	***n***	**Mean difference (SE)**	***t***	***p***
Bedtime (hh:mm)	Pre	22:58 (1:30)	135	1:22 (0:13)	6.18	**< 0.001** ^**^
	During	24:20 (2:35)				
Midpoint (hh:mm)	Pre	2:55 (1:35)	135	1:13 (0:10)	6.66	**< 0.001** ^**^
	During	4.09 (2:06)				
TIB (min)	Pre	468.8 (90)	135	9.63 (9.2)	1.05	0.295
	During	478.4 (104.3)				
nTST (min)	Pre	445.6 (90.8)	123	−7.36 (12)	−0.61	0.541
	During	438.3 (112)				
SE%	Pre	84 (15.2)	110	−9.48 (1.63)	−5.83	**< 0.001** ^**^
	During	74.5 (18.9)				
Nightmares/week	Pre-#	1.5 (2.5)	105	1.49 (0.26)	5.78	**< 0.001** ^**^
	During-#	3 (3.5)				
Naps/week	Pre-#	2.1 (2.3)	95	1.14 (0.3)	3.82	**< 0.001** ^**^
	During-#	3.2 (3.5)				

TIB, time-in-bed; nTST, nocturnal total sleep time; SE, sleep efficiency.

The bold values mean significant values, either *p* < 0.05 or *p* < 0.001.

^**^p < 0.001.

### Bivariate associations and regression analyses with ISI and sleep efficiency

Bivariate associations showed that the variables significantly related to ISI with *r* ≥ |0.3| or η^2^ ≥ 0.06 were sex, income, education, CES-D-10, SHPS subscales including arousal-associated behaviors, sleep scheduling behaviors, and sleep environment, changed sleep-wake routine, and CIS total score (Supplementary Tables 1, 2). Sleep efficiency was similarly associated with income, CES-D, SHPS two subscales (arousal-associated behaviors and sleep scheduling behaviors), changed sleep-wake routine, sleep efficiency before the pandemic, and CIS total score (Supplementary Tables 1, 2). Variables that were weakly associated with ISI (i.e., *r* < |0.3| or η^2^ < 0.06), and thus not included in the regression analyses, were race (η^2^ = 0.05) and the SHPS subscale of eating/drinking behaviors prior to sleep (*r* = 0.269). Sleep efficiency was similarly weakly associated with sex (η^2^ = 0.02), race (η^2^ = 0.03), education (η^2^ = 0.05), and the SHPS subscales of eating/drinking behaviors prior to sleep (*r* = −0.144) and sleep environment (*r* = −0.188).

The hierarchical multiple regression with ISI as outcome revealed that in model 1 (Table 3), sex, income (>$50 k vs. < $10 k USD), and education contributed significantly to the regression model [*F*_(4, 117)_ = 8.978, *p* < 0.001], and accounted for 23.5% of the variation. In model 2, sex and income (>$50 k vs. < $10 k USD) remained significantly associated after introducing CES-D-10, which explained an additional 22.8% of the variation, with this change in *R*^2^ being significant [*F*_(1, 116)_ = 49.107, *p* < 0.001]. The addition of SHPS subscales and the changed sleep-wake routine to the regression model 3 explained an additional 26.3% of the variation, with this increase in *R*^2^ also being significant [*F*_(6, 110)_ = 17.604, *p* < 0.001]. Finally, adding the CIS total score to the regression model 4 explained an additional 1.1% of the variation, and this change in *R*^2^ was significant [*F*_(1, 109)_ = 4.564, *p* = 0.035]. When all the independent variables were included in the final model, sex became a non-significant predictor. Household income and sleep environment interferences were negatively associated with ISI, and depressive symptoms, frequent arousal-associated activities, maladaptive sleep scheduling, and the impact of the COVID-19 pandemic on daily life were positively related to ISI. All the independent variables accounted for 73.7% of the variance in ISI.

**Table 3:** Multiple regression results for predictors hypothesized to be associated with the insomnia severity index (ISI) (*n* = 121).

**Predictors**	**Model 1**				**Model 2**				**Model 3**				**Model 4**			
	***b***	β	***t***	***p***	***b***	β	***t***	***p***	***b***	β	***t***	***p***	***b***	β	***t***	***p***
**Male** ^a^	−5.13	−0.05	−4.32	< 0.001	−3.22	−0.03	−3.11	0.002	−0.63	−0.01	−0.78	0.437	−0.47	−0.01	−0.59	0.556
**Income** ^b^																
10 k−49,999	−0.5	−0.01	−0.37	0.713	−0.7	−0.01	−0.62	0.54	−1.78	−0.02	−2.08	0.04	−1.67	−0.02	−1.98	0.05
≥50 k	−3.8	−0.04	−2.96	0.004	−2.5	−0.03	−2.28	0.024	−2.24	−0.03	−2.71	0.008	−2	−0.02	−2.44	**0.016** ^*^
**Education** ^c^																
Bachelor or above	−2.69	−0.03	−2.2	0.03	−1.26	−0.01	−1.2	0.233	−0.52	−0.01	−0.66	0.509	−0.5	−0.08	−0.03	0.521
CES-D-10					0.53	0.52	7.01	< 0.001	0.23	0.23	3.49	< 0.001	0.2	0.2	3.03	**0.003** ^*^
**SHPS**																
Arousal-associated behaviors									0.45	0.5	6.49	< 0.001	0.44	0.48	6.38	**< 0.001** ^**^
Sleep scheduling behaviors									0.24	0.25	3.71	0.001	0.23	0.23	3.54	**< 0.001** ^**^
Sleep environment									−0.17	−0.19	−3.16	0.002	−0.18	−0.2	−3.25	**0.002** ^*^
**Whether quarantine sleep-wake routine was changed and consistent with their personal preference** ^d^																
Yes, not consistent with their personal preference									2.91	0.03	2.45	0.016	2.73	0.03	2.33	**0.022** ^*^
Yes, somewhat more consistent with their personal preference									1.06	0.01	0.86	0.394	1.04	0.01	0.85	0.396
Yes, much more consistent with their personal preference									2.64	0.02	2.08	0.04	2.52	0.02	2.01	**0.047** ^*^
**CIS total**													0.18	0.12	2.14	**0.035** ^*^
*R* ^2^	0.235				0.462				0.726				0.737			
Δ*R*^2^	0.235				0.228				0.263				0.011			
*F* for Δ*R*^2^	8.978^**^				49.107^**^				17.604^**^				4.564^*^			
*F* for the model	8.978^**^				19.957^**^				26.464^**^				25.426^**^			

^a^Reference group: female.

^b^Reference group: < 10 k.

^c^Reference group: below bachelor.

^d^Reference group: no change and consistent with preference.

^*^p < 0.05.

^**^p < 0.001.

The bold values mean significant values, either *p* < 0.05 or *p* < 0.001.

For the dummy-coded predictors, β = b/sdy; for the numeric predictors, β = (b^*^sdx)/sdy.

The hierarchical multiple regression with sleep efficiency as outcome revealed that in model 1 (Table 4), income (>$50 k vs. < $10 k USD) contributed significantly to the regression model [*F*_(2, 106)_ = 8.207, *p* < 0.001], and accounted for 13.4% of the variation. In model 2, income (>$50 k vs. < $10 k USD) remained significantly associated with sleep efficiency after introducing CES-D-10, which accounted for an additional 9.8% of the variation, with this change in *R*^2^ being significant [*F*_(1, 105)_ = 13.349, *p* < 0.001]. The addition of two SHPS subscales, the changed sleep-wake routine due to the pandemic, and the sleep efficiency before the pandemic into the regression model 3 explained an additional 22.6% of the variation, and this change in *R*^2^ was also significant [*F*_(6, 99)_ = 6.859, *p* < 0.001]. Finally, adding the CIS total score to the regression model 4 explained an additional 1.2% of the variation, but this change in *R*^2^ was not significant [*F*_(1, 98)_ = 2.129, *p* = 0.148]. When all the independent variables were included in the final model, CES-D-10, arousal-associated behaviors and sleep scheduling behaviors, changed sleep-wake routine, and CIS became non-significant predictors. Household income and sleep efficiency before the pandemic were positively associated with sleep efficiency during the pandemic. Together these independent variables accounted for 46.9% of the variance in sleep efficiency.

**Table 4:** Multiple regression results for predictors hypothesized to be associated with sleep efficiency (SE) (*n* = 108).

**Predictors**	**Model 1**				**Model 2**				**Model 3**				**Model 4**			
	***b***	β	***t***	***p***	***b***	β	***t***	***p***	***b***	β	***t***	***p***	***b***	β	***t***	***p***
**Income** ^a^																
10 k−49,999	5.82	0.01	1.37	0.173	5.76	0.01	1.43	0.154	4.12	0.01	1.15	0.253	3.88	0.01	1.09	0.279
≥50 k	15.91	0.02	4	< 0.001	13.52	0.02	3.54	< 0.001	8.4	0.01	2.38	0.019	7.96	0.01	2.26	**0.026** ^*^
**CES-D-10**					−0.92	−0.32	−3.65	< 0.001	−0.22	−0.08	−0.78	0.435	−0.13	−0.04	−0.45	0.651
**SHPS**																
Arousal-associated behaviors									−0.46	−0.18	−1.71	0.09	−0.42	−0.16	−1.56	0.123
Sleep scheduling behaviors									−0.5	−0.18	−1.86	0.067	−0.46	−0.17	−1.71	0.091
**Whether quarantine sleep-wake routine was changed and consistent with their personal preference** ^b^																
Yes, not consistent with their personal preference									−3.03	−0.004	−0.61	0.542	−2.48	−0.004	−0.5	0.617
Yes, somewhat more consistent with their personal preference									2.70	0.003	0.52	0.602	2.83	0.003	0.55	0.583
Yes, much more consistent with their personal preference									−3.69	−0.004	−0.69	0.49	−3.4	−0.004	−0.64	0.522
Sleep efficiency before the COVID-19 pandemic									0.42	0.34	4.11	< 0.001	0.4	0.32	3.91	**< 0.001** ^**^
**CIS total**													−0.51	−0.13	−1.46	0.148
*R* ^2^	0.134				0.232				0.457				0.469			
Δ*R*^2^	0.134				0.098				0.226				0.012			
*F* for Δ*R*^2^	8.207^**^				13.349^**^				6.859^*^				2.129			
*F* for the model	8.207^**^				10.558^**^				9.271^**^				8.652^**^			

^a^Reference group: < 10 k.

^b^Reference group: No change and consistent with preference.

^*^p < 0.05.

^**^p < 0.001.

The bold values mean significant values, either *p* < 0.05 or *p* < 0.001.

For the dummy-coded predictors, β = b/sdy; for the numeric predictors, β = (b^*^sdx)/sdy.

## Discussion

To the best of our knowledge, the present study is one of few to investigate changes in sleep patterns and insomnia symptoms during the early wave of the COVID-19 pandemic among parents and caregivers of young children. Our findings indicated that more than half of parents and caregivers of young children experienced clinical levels of insomnia symptoms (51.5%) and poor sleep efficiency (65.8%), in line with other parental studies (Zreik et al., 2020; Wearick-Silva et al., 2022). Consistent with our original hypothesis, greater severity of insomnia symptoms was associated with lower income, greater depressive symptoms, more frequent arousal-associated activities and maladaptive sleep scheduling behaviors, less frequent sleep environment interferences, alterations in sleep-wake routine due to the pandemic, and greater impact of the COVID-19 pandemic on daily life. Lower sleep efficiency was associated with lower income and poorer sleep efficiency before the pandemic.

Low income was found to be strongly associated with insufficient sleep (Grandner et al., 2015), increased sleep latency (Lauderdale et al., 2006), and poorer sleep efficiency (Lauderdale et al., 2006; Friedman et al., 2007) before the pandemic. U.S. Bureau of Economic Analysis reported that at the start of the COVID-19 pandemic, personal income decreased by 413.8 billion USD (Garrison et al., 2022). Further, more than 40% of parents lost work-related income due to the COVID-19 outbreak (Karpman et al., 2020). Nearly 24 million were parents with young children (< 6 y) (Karpman et al., 2020). The reduced income during the pandemic was associated with greater depressive symptoms (Hibel et al., 2021), which may serve as a mediator between decreased income and both greater insomnia and lower sleep efficiency (Friedman et al., 2007; El-Sheikh et al., 2015). Additionally, the pandemic impacted individuals' health (Sherman et al., 2020). Health may also mediate the relationship between lower income and both shorter sleep duration and lower sleep efficiency (Friedman et al., 2007; Stamatakis et al., 2007).

In line with other studies during the pandemic (Salari et al., 2020), caregivers' depression prevalence was high (59.2%). A study monitoring 85,328 childcare centers in the U.S during the pandemic, indicated that April and May 2020 experienced the highest rate of childcare center closures between 59 and 70% (Lee and Parolin, 2021). A greater caregiving burden due to daycare closures (Russell et al., 2020), potentially compromised time for caregivers' self-care and emotional health. Additionally, fewer daycare attendance days may increase parental stress (American Psychological Association, 2020) and amplify parents' negative childcare feelings, such as being “irritated” and “tempted to hit my child” (Shinomiya et al., 2021). Finally, isolated individuals with limited social support had a higher risk for depression (Grey et al., 2020). These factors may contribute to greater depressive symptoms among parents (Lyttelton et al., 2020; Collins et al., 2021; Johnson et al., 2021). Similar to pre-pandemic (Morawetz, 2003), greater depression was also associated with greater insomnia symptoms during the pandemic (Bartoszek et al., 2020; Ye et al., 2020; Pizzonia et al., 2021).

Similar to other studies (Bacaro et al., 2020), poor sleep hygiene behaviors were associated with greater insomnia symptom severity. Work-life interruptions were related to poor arousal-associated behaviors (Gorgoni et al., 2021). During the pandemic, parents experienced decreased working hours compared with the pre-pandemic (Kochhar, 2020; Collins et al., 2021), and parents of children (< 5 years) were reported as having the least working hours (Kochhar, 2020). The reduced working hours may alter their sleep/wake routine and increase non-sleep promoting activities in the bedroom. Further, the pandemic-related disruptions created new conflicts due to partners' insufficient caregiving support (Calarco et al., 2020). The additional family conflicts may have led to greater cognitive arousal and unpleasant conversations prior to and during the sleep period. All these poor arousal-associated behaviors may contribute to greater insomnia severity.

In this study, nearly 90% of participants reported their sleep-wake routine was changed. They went to bed later during the pandemic than pre-pandemic (24:20 vs. 22:58) and the sleep midpoint was also delayed, similar to other studies (Bacaro et al., 2021; Di Giorgio et al., 2021). Altered sleep schedules may be partly driven by their children's sleep timing. For children who attended a nursery school for two or fewer days a week during the pandemic, both caregivers and the children had later wake-up times when compared to those of children attending a nursery school longer (Shinomiya et al., 2021). Consistent sleep schedules are essential to align caregivers' sleep-wake schedules to their endogenous circadian rhythm, allowing them to pursue better sleep quality (Gellis et al., 2014). Poor sleep scheduling behaviors and altered sleep-wake routines during the pandemic contribute to caregivers' greater insomnia severity.

Inconsistent with other studies (Desaulniers et al., 2018; Holbert et al., 2021), our findings indicated that greater sleep environment interferences were associated with less insomnia severity. Why the present study found an opposing association is unclear. Considering the moderate positive association between sleep environment and insomnia severity, and the strong positive associations between other SHPS scales and insomnia severity (Supplementary Table 2), suppression analysis was further conducted (Pandey and Elliott, 2010). We found that the involved suppressor variables included CES-D-10, arousal-associated behaviors, sleep scheduling behaviors, whether quarantine sleep-wake routine was changed and consistent with their personal preference, and CIS total (Supplementary Table 3). This suppression effect may be due to an underspecified model (Garbin, 2011).

COVID-related disruptions in daily life were positively related to worse insomnia symptoms, similar to other studies (Cellini et al., 2020). Family's daily routines were affected (Toran et al., 2021), including working at irregular times to meet childcare demands and limited social life (Cox and Olatunji, 2021). Around 1/4 of parents with children aged < 6 y reported food insecurity in their households (Karpman et al., 2020). The limited access to secure food threatened family health including sleep. Further, reduced access to medical care and mental healthcare might also impact parents' health and daily life (Sherman et al., 2020). Additionally, the loss of in-person contacts reduced caregivers' social support from extended family and friends. They struggled to manage several responsibilities as caregivers, providers, and educators together (Adams, 2020). Finally, family discord also rapidly increased during the pandemic (Campbell, 2020; Calvano et al., 2021). Some parents described parent-child relationships negatively in terms of conflicts, communication struggles, and boredom (Öngören, 2021). The disrupted daily life is more likely to be linked to an inconsistent connection with circadian entrainment cues and a weak relationship between bedtime and sleep. These in turn may relate to disrupted sleep and insomnia (Cox and Olatunji, 2021).

We acknowledge the presence of limitations in the current study. First, participants in this online study were recruited via social media. This might introduce a selection bias favored to female-dominated and educated populations who were using the internet frequently. Second, this was a cross-sectional study. Insomnia was not assessed prior to the pandemic. Third, the sample size of this subsample was relatively small. Fourth, as predictors of parental sleep, we did not assess the severity of young children's sleep disturbances and the co-morbidities of the children (e.g., autism spectrum disorder/attention-deficit/hyperactivity disorder or other medical conditions). Sleep disturbances in young children are highly prevalent. Parental sleep can be affected by the sleep patterns of their young child (Sinai and Tikotzky, 2012; Rönnlund et al., 2016; Staples et al., 2019). Lastly, we did not assess the specific changes to childcare during the pandemic. Parent sleep and mood may have been differentially impacted.

## Conclusion

The insomnia symptoms were highly prevalent among parents and caregivers of young children. Sleep patterns, including sleep efficiency, markedly deteriorated from the patterns prior to the pandemic. The identified predictors associated with greater insomnia symptom severity and poor sleep efficiency included lower household income, greater depressive symptoms, poor sleep hygiene behaviors, altered sleep-wake routine, poor sleep efficiency before the pandemic, and greater COVID-related disruptions in daily life. Family-level and individual-level interventions are needed. First, additional childcare support for parents and caregivers to relieve caregiving stress should be provided. Second, mHealth-related approaches are generally accessible and can be practical interventions during pandemics. Third, public health education about promoting healthy sleep hygiene behaviors is also needed.

## Data availability statement

The raw data supporting the conclusions of this article will be made available by the authors, without undue reservation.

## Ethics statement

The studies involving human participants were reviewed and approved by the Institutional Review Board of Arizona State University. The patients/participants provided their written informed consent to participate in this study.

## Author contributions

Conceptualization and methodology: NJ and MP. Data analysis, review, and editing: NJ, MP, and KP. Original draft preparation: NJ. Supervision: MP. All authors have read and agreed to the published version of the manuscript.
