# Supplementary material for: Impact of COVID-19 pandemic on insomnia and sleep efficiency in parents and caregivers of young children
Source: Front Sleep. 2023 Jul 4;2:1212784. doi: 10.3389/frsle.2023.1212784 (PMC12713991; doi:10.3389/frsle.2023.1212784)
Supplement: Supplementary file 1 [file Table_1.docx]

Supplementary Material

**Impact of COVID-19 Pandemic on Insomnia and Sleep Efficiency in Parents and Caregivers of Young Children**

**Nana Jiao; Keenan A. Pituch; Megan E. Petrov^*^**

Edson College of Nursing and Health Innovation, Arizona State University, Phoenix, AZ, USA

**^*^Correspondence:**

Megan E. Petrov, PhD

Associate Professor,

Edson College of Nursing and Health Innovation

Arizona State University

500 N. 3^rd^ Street

Phoenix, AZ 85004

[Megan.Petrov@asu.edu](mailto:Megan.Petrov@asu.edu)

**Supplementary Tables**

**Supplementary Table 1. Comparisons of Insomnia and Sleep Efficiency among different socio-demographic subgroups**

|  |  | | | | Insomnia | | | | | | | | | | | | | | | | | | | | | | | | | | |  | | Sleep Efficiency | | | | | | | | | | | | | | | | | | | | | | | | | | | | | | | | | | | | | | | | | | | | | |
| --- | --- | --- | --- | --- | --- | --- | --- | --- | --- | --- | --- | --- | --- | --- | --- | --- | --- | --- | --- | --- | --- | --- | --- | --- | --- | --- | --- | --- | --- | --- | --- | --- | --- | --- | --- | --- | --- | --- | --- | --- | --- | --- | --- | --- | --- | --- | --- | --- | --- | --- | --- | --- | --- | --- | --- | --- | --- | --- | --- | --- | --- | --- | --- | --- | --- | --- | --- | --- | --- | --- | --- | --- | --- | --- | --- | --- | --- | --- | --- |
|  |  | | | M (SD) | | | | | | | | | | | | F | | | | | | | p | η^2^ M (SD) | | | | | | | | | | | | | | | | | | | | | | | | | | | | | F | | | | p | | | | | | | | | | | | | η^2^ | | | | | | | | | |
| Sex | | Female | | | | 11.85 (6.48) | | | | | | | | | | | 17.901 | | | | | **<0.001**** | | | | **0.12** | | | | | | | 73.31 (18.73) | | | | | | | | | | | | | | 2.035 | | | | | | | | | | | | | | 0.156 | | | | | | | | | | | 0.02 | | | | | | | |
|  | | Male | | | | 6.9 (5.54) | | | | | | |  | | | | | | | | | |  | | | | | | | | | | | | |  | | | | | | 78.87 (18.18) | | | | | | | | | | | | | |  | | | | | | | | | | |  | | | | | | | | | |  | | |
| Household  Income | | <$10,000 | | | | 11.98 (6.92) | | | | | | | | | | | 4.439 | | | | | | **0.014*** | | **0.06** | | | | | | | | | | | | | | | | | | 66.58 (19.64) | 8.839 | | | | | | | | | | | | | | | **<0.001**** | | | | | | | | | | | | | | | **0.14** | | | | | |
|  |  | $10,000-$49,999 | | | | 10.97 (6.18) | | | | | | | | | | | | |  | | | | | | | | |  | | | | | | | | | | | 73.02 (16.69) | | | | | | |  | | | | | | | | | | | | | | | |  | | | |  | | | | | | | | | |  | | | |
|  |  | ≥$50,000 | | | | 8.22 (5.96) | | | | | | | | | | | | |  | | | | | | | | |  | | | | | | | | | | | 82.61 (16.37) | | | | | | |  | | | | | | | | | | | | | | | |  | | | |  | | | | | | | | | |  | | | |
| Race | | Asian | | | | 10.78 (6.27) | | | | | | | | | | | 0.456 | | | | | | 0.5 | | 0.003 | | | | | | | | | | | | | 70.54 (20.82) | | | | | | | | | | | 3.835 | | | | | | | | | 0.053 | | | | | | | | | | | | | | | 0.03 | | | | | | |
|  | | White or others | | | | 10 (6.8) | | | |  | | | | | | | | |  |  | | | | | | | | | | | | | | | | | | | | 77.46 (16.9) | | | | | |  | | |  | | | | | | | | | | | | |  | | | | | |  | | | | | | | | | | | |
| Education | | Below bachelor | | | | 13.19 (7.99) | | | | | | | | | 9.682 | | | | | | | | **0.002*** | | | | **0.07** | | | | | | | | | | | 68.61 (18.89) | | | | | | | | | | | 5.437 | | | | | | | | | | | | | | | **0.021*** | | | | | | | 0.05^a^ | | | | | | | | |
|  | | Bachelor or above | | | | 9.35 (5.7) | | | | | | | |  | | | |  | | |  | | | | | | | | |  | | | | | | | | | | | | | 77.31 (18.09) | | | | | | |  | | | | |  | | | | | | | | | | |  | | | | | | | | |  | | | | |
| Changed sleep-wake routine | | Yes, not consistent with their preference | | | | 13.13 (6.02) | | | | | | **9.442** | | | | | | | | | | | **<0.001**** | | | | | | **0.19** | | | | | | | | 70.61 (19.95) | | | | | | | | | | | **2.865 0.04*** | | | | | | | | | | | | | | | | | | | | | | | | | **0.08** | | | | | | |
|  | | | Yes, somewhat more consistent | | | | 9.21 (4.66) | | | |  | | | | | | | | | | | | | | | | | | | |  | | | | | | | | | | 80.26 (12.28) | | | |  | | | | | | |  | | | | | | | |  |  |  |  |  |  |  |  |  |  |  |  |  |  |  |  |  |  |  |  |
|  |  |  | Yes, much more consistent | | | | | 8.86 (8.69) |  | | | | | | | | | | | | | | | | | | | | | | | | | | 75.55 (21.4) | | | | | | | | | | | | | | | | | | |  | | | | | | | | |  | | | | | |  | | | | | | | | |  |  |
|  |  |  | No change and consistent with preference | | | | | 4.73 (3.03) |  | | | | | | | | | | | | | | | | | | | | | | | | | | 84.84 (13.27) | | | | | | | | | |  | | | | | |  | | | | | | | | | | | | | |  | | | | | | | | | | | | | | |

Note: *p<0.05, **p<0.001; ^a^ Dropped when entering the hierarchical multiple regressions, due to η^2^ < 0.06.

**Supplementary Table 2. Correlation matrix of Insomnia and Sleep Efficiency during the pandemic**

|  | Age | CES-D-10 | SHPS Subscale: Arousal-associated behaviors | SHPS Subscale: Eating/drinking behaviors | SHPS Subscale: Sleep scheduling | SHPS Subscale: Sleep environment | Sleep Efficiency before the pandemic | | CIS |
| --- | --- | --- | --- | --- | --- | --- | --- | --- | --- |
| Insomnia | -0.149 | **0.624^**^** | **0.752^**^** | 0.269^**a^ | **0.634^**^** | **0.320^**^** | |  | **0.503^**^** |
| Sleep Efficiency | 0.081 | **-0.373^**^** | **-0.465^**^** | -0.144 | **-0.412^**^** | -0.188^*a^ | | **0.514^**^** | **-0.408^**^** |

Note: ^**^Correlation is significant at the 0.01 level (2-tailed); ^*^Correlation is significant at the 0.05 level (2-tailed); ^a^ Dropped when entering the hierarchical multiple regressions, due to correlation coefficient r <|0.3|.

**Supplementary Table 3.** Multiple regression to explore the suppressor variables (n=136)

| **Predictors** | | **Model 1** | | | | | | **Model 2** | | | | | |
| --- | --- | --- | --- | --- | --- | --- | --- | --- | --- | --- | --- | --- | --- |
|  |  | b | | β | t | p | | b | β | t | | p | |
| **Male^1^** | | -5.13 | | -0.05 | -4.45 | | <0.001 | -4.65 | -0.05 | | -4.04 | <0.001** |  |
| **Income^2^** | |  | |  |  | |  |  |  | |  |  | |
| 10k-49,999 | | -0.5 | | -0.004 | -0.38 | | 0.705 | -0.14 | -0.002 | | -0.11 | 0.914 | |
| ≥50k | | -3.8 | | -0.04 | -3.05 | | 0.003 | -3.1 | -0.03 | | -2.45 | 0.016* | |
| **Education**^3^  Bachelor or above | -2.69 | | | -0.03 | -2.26 | | 0.025 | -2.28 | -0.02 | | -1.93 | 0.056 | |
| **Sleep environment** | | |  |  |  |  | | **0.17** | **0.19** | | **2.24** | **0.027*** | |
| R^2^ | | | 0.235 | | | | | 0.265 | | | | | |
| ΔR^2^ | | | 0.235 | | | | | 0.03 | | | | | |
| F for ΔR^2^ | | | 9.515** | | | | | 5.020** | | | | | |
| F for the model | | 9.515** | | | | | | 8.863** | | | | | |

Note: ^1^ reference group: female; ^2^ reference group: < 10k; ^3^ reference group: below bachelor; *p<0.05, **p<0.001; For the dummy-coded predictors, β = b/sdy; for the numeric predictors, β = (b*sdx)/sdy.
